# Two New Species of Batrisini (Coleoptera: Staphylinidae: Pselaphinae) from Nanling Mountain Area, China †

**DOI:** 10.3390/insects13020119

**Published:** 2022-01-24

**Authors:** Wen-Xuan Zhang, Zi-Wei Yin

**Affiliations:** Laboratory of Systematic Entomology, College of Life Sciences, Shanghai Normal University, 100 Guilin Road, Xuhui District, Shanghai 200234, China; zhangwx@protonmail.com

**Keywords:** *Batrisceniola*, *Physomerinus*, new species, Nanling, China, taxonomy

## Abstract

**Simple Summary:**

The Nanling Mountains are the largest mountain range and an important physical geographical boundary in Southeast China, but their insect diversity has not been sufficiently documented up to now. Through recent investigations of the local staphylinid fauna at Nanling, more than 4500 adult pselaphine beetles were collected and most of the samples belong to the highly diverse tribe Batrisini. Here, two new species of the genera *Batrisceniola* and *Physomerinus*, both poorly represented in the Chinese pselaphine fauna, are described.

**Abstract:**

Two new species of the *Batrisocenus* complex of genera, e.g., *Batrisceniola nanlingensis* sp. nov. and *Physomerinus clavipes* sp. nov., are described, diagnosed, and illustrated from the Nanling Mountain Area. Both represent a second species of the respective genus from the Chinese mainland.

## 1. Introduction

As one of the 32 inland territorial and aquatic biodiversity conservation priority areas [1], Nanling Mountain Area is the largest mountain system as well as an important geographical boundary in southern China. It is also the largest oasis near a latitude of 25 degrees north and harbors a high diversity of plants and animals [2]. Through a joint project led by Prof. Xing-Ke Yang, we had an opportunity to collect more than 4500 adult pselaphine beetles in most conservation areas of Nanling. Approximately 68% of the sampled specimens belong to the diverse tribe Batrisini. In contrast to this number, only 16 species classified in 9 genera of the tribe are currently known to occur in Nanling, which indicates that the true diversity of this group still remains poorly documented.

The Oriental genus *Batrisceniola* Jeannel, 1958 is represented by four species from China (1 sp.) and Japan (3 spp.): *B. dissimilis* (Sharp, 1874), *B. semipunctulata* (Raffray, 1909), *B. hiranoi* Nomura, 1991, and *B. fengtingae* Yin and Li, 2014 [3,4,5,6,7]. Members of *Batrisceniola* are characterized by the abdominal tergite 4 (VII) bearing a median bunch of erect setae in both sexes. On the other hand, the genus *Physomerinus* Jeannel, 1952, defined by the presence of sexual characters on male metafemora and a constricted basal capsule of the aedeagus, includes 11 species distributed in China (3 spp.), Japan (including the Ryukyu Islands) (3 spp.), Indonesia (3 spp.), Thailand (2 spp.), Vietnam (1 sp.), Malaysia (1 sp.), Borneo (1 sp.), Singapore (1 sp.), New Guinea (1 sp.), and Myanmar (1 sp.) [3,6,8,9,10,11,12,13,14,15,16,17]. An examination of the material collected from Nanling revealed one new species of each genus, which are formally described in the present paper.

## 2. Materials and Methods

The material treated in this study is housed in the Insect Collection of Shanghai Normal University (SNUC). The label data of the material are quoted verbatim.

Dissected parts were preserved in Euparal on plastic slides that were placed on the same pin as the specimens. The habitus images were taken using a Canon 5D Mark III camera (Ōita, Japan) in conjunction with a Canon MP-E 65 mm f/2.8 1-5X Macro Lens (Ōita, Japan), and a Canon MT-24EX Macro Twin Lite Flash (Ōita, Japan) was used as the light source. Images of morphological details were produced using a Canon G9 camera (Zhuhai, China) mounted to an Olympus CX31 microscope (Ina-Shi, Japan) under transmitted light. Zerene Stacker (version 1.04) was used for image stacking. All images were optimized and grouped into plates using Adobe Photoshop CS5 Extended.

The abdominal tergites and sternites are numbered following Chandler (2001) [18] in Arabic (starting from the first visible segment) and Roman (reflecting true morphological position) numerals, e.g., tergite 1 (IV), or sternite 1 (III).

Measurements were taken as the following: the total body length was measured from the anterior margin of the clypeus to apex of the abdomen; head length was measured from the anterior margin of the clypeus to head base, excluding occipital constriction; head width was measured across the eyes; the length of the pronotum was measured along the midline, width equals its maximum width; the length of the elytra was measured along the suture; the width is the maximum width across both elytra; the length of the abdomen is the length of the dorsally exposed part of the abdomen along the midline; the width is abdomen’s maximum width.

## 3. Results

**Genus *Batrisceniola* Jeannel, 1958**

*Batrisceniola* Jeannel, 1958: 65. Type species: *Batrisus dissimilis*, Sharp, 1874 (original designation).

Jeannel placed this genus in his fifth division of Batrisina, and defined it mainly based on the male frons bearing a pair of large excavations [4], a criterion apparently of little phylogenetic importance beyond species level. In a recent work, Nomura redefined *Batrisceniola* on the basis of the large clypeus, and tergite 4 (VII) of the abdomen with a median bunch of setae in both sexes [6]. *Batrisceniola* resembles *Arthromelodes*, Jeannel, 1954, in the similar configurations of the aedeagus and female genitalia, but can be readily separated by the tergal setose brush. The first and the only Chinese congener, *B*. *fengtingae*, Yin and Li, was described in 2014 [7], which possesses a simple head and a modified tergite 1 (IV).

***Batrisceniola nanlingensis* Zhang & Yin, sp. nov.**

**Type material** (20 exx.)**.** Holotype: CHINA: ♂, ‘China: Guangdong, Shaoguan, Ruyuan, Nanling N. R., Pubuqun (Falls Scenic Zone), 24°54′9″ N, 113°2′53.8″ E, 660–850 m, 3.V.2021, sifting, Hu, Lin, Zhou and Li leg.’ (SNUC). Paratypes: CHINA: 2 ♀♀, same locality as holotype (SNUC); 2 ♂♂, same locality as holotype except ‘Xiaohuangshan, 24°53′44.7″ N, 113°1′26.9″ E, 1270–1570 m, 2.V.2021,’ (SNUC); 1 ♂, 2 ♀♀, same locality as holotype except ‘Qinshui Valley, 24°55′42.9″ N, 113°0′59.05″ E, 680–780 m, 5.V.2021,’ (SNUC); 10 ♂♂, same locality as holotype except ‘Guang-dong-di-yi-feng, 24°55′29″ N, 112°59′31″ E, 1538–1784 m, 28.VI.2020, Xia, Zhang, Yin, Lin leg.’ (SNUC); 2 ♂♂, ‘China: Guangdong Prov. Ruyuan County, Nanling N. R. Sta. alt. 1100 m 14, VIII, 2008, QI N and YIN Z-W leg.’ (SNUC).

**Figure 1:**
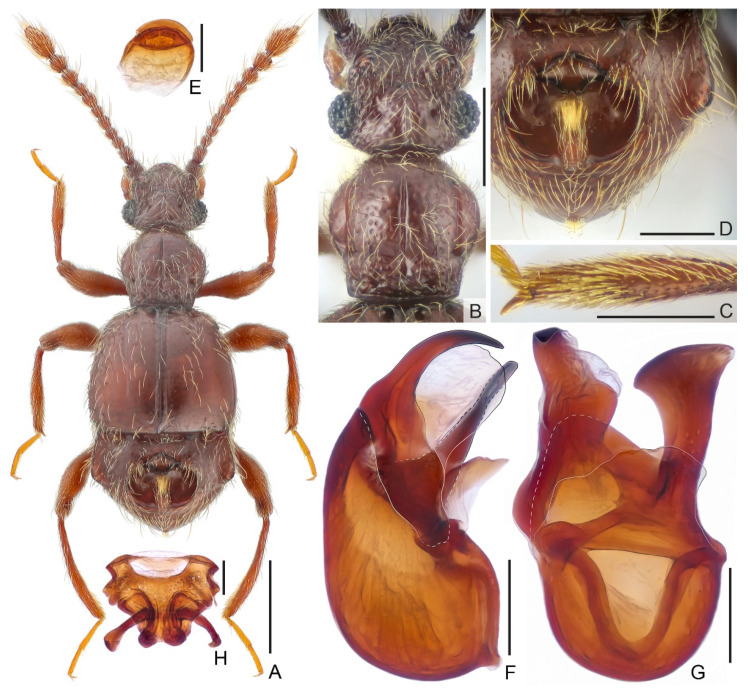
*Batrisceniola nanlingensis* sp. nov. ((**A**–**G**). Male. (**H**). Female). (**A**) Dorsal habitus; (**B**) Head and pronotum; (**C**) mesotibia; (**D**) Tergite 1–4 (IV–VII); (**E**) sternite 7 (IX); (**F**,**G**) Aedeagus, in lateral (**F**) and ventral (**G**) view; (**H**) Female genitalia. Scale bars: 0.5 mm in (**A**); 0.3 mm in (**B**); 0.2 mm in (**C**,**D**); 0.1 mm in (**E**–**H**).

**Diagnosis.***Male.* Body length is approximately 2.0 mm. The head is sub-rectangular, with indistinct vertex sulcus connecting large vertexal foveae, with a thin mediobasal carina extending from head base anteriorly and passing the level of the anterior margin of the eyes; antennae elongate, lacking distinct club, antennomere 11 largest. Pronotum with distinct median sulcus and pair of lateral longitudinal sulci, with broad transverse antebasal impression. Discal striae of elytra shallow, extending posteriorly to apical 1/3 of elytral length, with small, angulate humeral prominence. Protibia with apical tuft of setae at mesal margin; mesotibia with distinct apical spine. Tergite 4 (VII) with a large central cavity and expanded laterally. Aedeagus strongly asymmetric, stout; ventral stalk of median dilated at apex; dorsal lobe in lateral view narrowing toward apex, with broad membranous ventral lamella; parameres fused to broad membrane. *Female.* Body length approximately 2.0–2.1 mm; antennae slightly shorter; legs lacking apical tuft of setae or spine; genital complex as in Figure 1H.

**Description.***Male.* Body (Figure 1A) length 1.98–2.03 mm; color reddish-brown, tarsi and mouthpart lighter.

Head (Figure 1B) sub-rectangular, slightly wider than long, length from anterior margin of clypeus to head base 0.42–0.43 mm, width across eyes 0.48 mm; vertex shallowly and roughly punctate at middle, punctation much coarser posterior antennal tubercles, with large asetose vertexal foveae (dorsal tentorial pits), with indistinct U-shaped sulcus connecting foveae, mediobasal carina thin, extending from head base anteriorly and passing level of anterior margin of eyes, approaching transverse, arcuate carina between antennal tubercles, lateral carina extending from base to posterior margin of antennal tubercle; posterolateral margin round; lacking frontal-clypeal ridge, clypeus with carinate and moderately raised anterior margin; ocular-mandibular carina complete, distinct. Venter with rough surface; small gular foveae (posterior tentorial pits) in shared round opening, with faint median carina extending from opening anteriorly to mouthpart. Compound eyes prominent, each composed of approximately 30 ommatidia. Antenna elongate, length 1.01–1.03 mm, lacking distinct club; antennomere 1 thick, subcylindrical, with dense setae on anterolateral margin, 2 elongate, 3–5 each slightly elongate, 6 smallest, 7 slightly wider than 5, 8 moniliform, much smaller than 7, 9–11 each moderately enlarged, 11 largest, approximately as long as antennomeres 9 and 10 combined.

Pronotum (Figure 1B) approximately as long as wide, length 0.45 mm, width 0.47–0.48 mm, widest at middle; sides rounded, convergent basally, disc slightly convex, with shallow, rough punctures, median longitudinal sulcus with carinate sides, with pair of lateral longitudinal sulci; broad antebasal sulcus connecting large, asetose lateral antebasal foveae; with small outer and inner pair of basolateral foveae. Prosternum with anterior part shorter than coxal part, with large lateral procoxal foveae; thin hypomeral ridge extending from base to middle of anterior part; margins of coxal cavities obviously carinate.

Elytra wider than long, length 0.69–0.7 mm, width 0.81–0.86 mm; each elytron with two moderately large, asetose basal foveae; thin discal stria extending posteriorly from outer basal fovea to apical 1/3 of elytral length; with small, angulate humeral prominence, subhumeral fovea present, carinate marginal stria extending from fovea to posterior margin of elytron.

Mesoventrite short, demarcated from metaventrite by transverse carina; median mesoventral foveae widely separated, in shared opening, large lateral mesoventral foveae forked internally, marginal stria complete. Metaventrite slightly prominent, weakly impressed at middle, with well-developed lateral mesocoxal and two lateral metaventral foveae; posterior margin with small and narrow split at middle.

Legs elongate; protibia with apical tuft of setae, protrochanter with small ventral denticle near apex, procoxa with long seta at ventral margin; mesotibia (Figure 1C) with distinct apical spine.

Abdomen widest at mesal margins of tergite 1 (IV), length of abdomen 0.64–0.65 mm, width 0.79–0.82 mm. Tergite 1 (IV) (Figure 1D) approximately three times as long as 2–4 (V–VII) combined, expanded on both sides, widest near middle; discal carina short but distinct, thin basal sulcus separated by mediobasal and large basolateral foveae, lacking inner marginal carina, outer one complete, with large, deep oval cavity at middle of tergite, anterior margin of cavity ridged, posterior margin with large, setiferous oval nodule; tergite 2 (V) as long as 3 (VI), 4 (VII) longer than tergites 2 and 3 combined, posterior half of tergite 4 (VII) with bunch of erect setae at middle; tergite 5 (VIII) semicircular, transverse, posterior margin roundly emarginate at middle. Sternite 2 (IV) with one pair of mediobasal and one pair of basolateral foveae, with short lateral carina; midlength of sternites 3–5 (V–VII) short, each with one pair of small basolateral foveae; sternite 6 (VIII) transverse, posterior margin broadly emarginate; sternite 7 (IX) (Figure 1E) semi-membranous, apical portion rounded and more strongly sclerotized.

Aedeagus (Figure 1F,G) 0.37 mm long, strongly asymmetric; median lobe with rounded basal capsule and roundly triangular basal foramen, ventral stalk elongate, broadened at apex; dorsal lobe elongate, dorsoventrally widest at apical third, then narrowing apically, strongly curved to ventral side at apex, with broad membranous ventral lamella; parameres fused to single, broad membranous structure.

*Female.* Similar to male in external morphology; antenna slightly shorter; protibia lacking long apical tuft of setae, protrochanter lacking ventral denticle, mesotibia lacking apical spine; each compound eye composed of approximately 25 ommatidia; tergite 1 (IV) without median cavity. Measurements (as for male): body length 2.03–2.06 mm; length/width of head 0.43–0.44/0.49–0.50 mm, pronotum 0.45–0.47/0.48 mm, elytra 0.62–0.63/0.80–0.81 mm; abdomen 0.64–0.68/0.75–0.78 mm; length of antenna 0.95–0.97 mm; maximum width of genitalia (Figure 1H) 0.35 mm.

**Comparative notes.** The new species morphologically resembles the Japanese *Batrisceniola semipunctata* (Raffray, 1909) in the presence of a large cavity on tergite 1 (IV), as well as the shape of the aedeagus. They can be separated by the different structure of the cavity, the lack of lateral circular setiferous patches of tergite 1 (IV), and the dorsal lobe of the aedeagus lacks a subapical protuberance in *B*. *nanlingensis* sp. nov.

**Distribution.** China: Guangdong.

**Etymology.** The new species is named after its type locality, i.e., Nanling National Forest Park.

**Key to species of *Batrisceniola* from China** (male)

1 Tergite 1 (IV) with small cavity located near posterior margin [Reference [7]: Figure 1F], lateral margins rounded; aedeagus with extended ventral stalk apically narrowing and greatly bent [Reference [7]: Figure 1K] ………… *B. fengtingae* Yin and Li 2014
- Tergite 1 (IV) with large cavity located near middle (Figure 1A,D), lateral margins expanded; aedeagus with short ventral stalk apically greatly dilated (Figure 1G) ………………………………………………………………………*B. nanlingensis* sp. nov.

**Genus *Physomerinus*** **Jeannel, 1952**

*Physomerinus* Jeannel, 1952b: 96. Type species: *Batrisus* *septemfoveolatus* Schaufuss, 1877 (original designation).

Similar to many Asian genera created by Jeannel which are often characterized by male sexual characters, the genus *Physomerinus* is defined based on the sexually modified hind femora of the male, as well as by the aedeagus with a constricted basal capsule [6]. The genus *Btriscenaulax* Jeannel, with five species from Japan, also has the aedeagus with a small basal capsule, and under current definition is separated from *Physomerinus* by the modified protibiae and tergite 1 (IV) in the male. The relationship between these two genera is evidently close, and needs to be further investigated.

***Physomerinus*** ***clavipes*** **Zhang & Yin, sp. nov.**

**Type material** (2 exx.)**.** Holotype: CHINA: ♂, ‘China: Guangxi, Guilin, Huaping N. R., 25°37′39.83″ N, 109°54′20.23″ E, 780 m, 18.VIII.2020, streamside, Qiu Lu leg.’ (SNUC). Paratype: CHINA: 1 ♀, same locality as holotype (SNUC).

**Figure 2:**
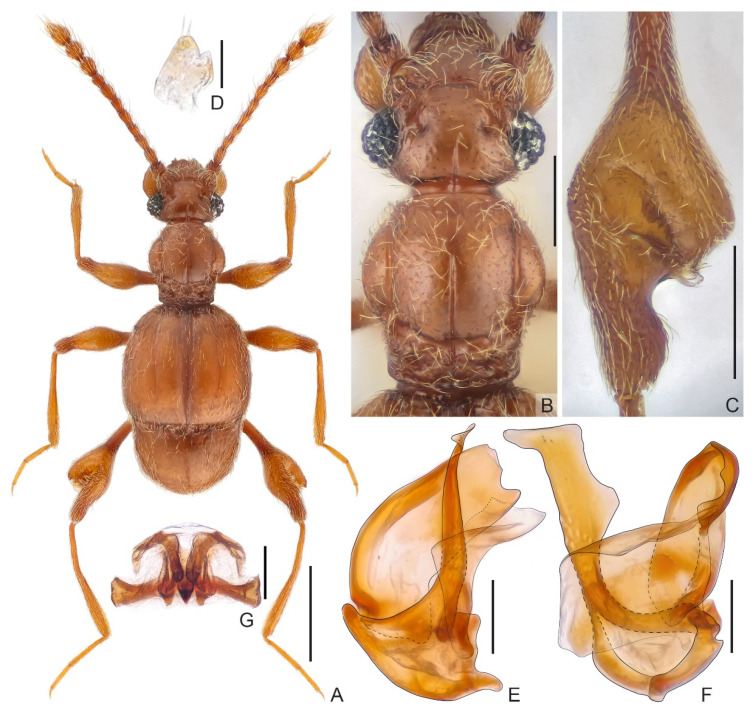
*Physomerinus clavipes* sp. nov. ((**A**–**F**). Male. (**G**). Female). (**A**) Dorsal habitus; (**B**) Head and pronotum; (**C**) metafemur; (**D**) sternite 7 (IX); (**E**,**F**) Aedeagus, in lateral (**E**) and ventral (**F**) view; (**G**) Female genitalia. Scale bars: 0.5 mm in (**A**); 0.2 mm in (**B**,**C**); 0.1 mm in (**G**); 0.05 mm in (**E**,**F**); 0.03 mm in (**D**).

**Diagnosis.***Male*. Body length approximately 1.8 mm. Head sub-rectangular, with distinct vertex sulcus connecting large, asetose vertexal foveae, with thin mediobasal carina extending from head base to anteriorly beyond level of posterior margin of eyes, antennae elongate, lacking distinct club, antennomere 11 largest. Pronotum with median sulcus and pair of lateral longitudinal sulci, with broad antebasal sulcus. Discal striae extending posteriorly from outer basal fovea to apical 1/5 of elytral length with small, angulate humeral prominence. Protibia with apical tuft of setae; lateral margin of metafemur strongly swollen. Aedeagus strongly asymmetric; median lobe stout, ventral stalk dorsoventrally broadened at middle and apex; dorsal lobe in lateral view broad; parameres fused to broad membrane. *Female*. Body length approximately 1.9 mm; antennae slightly shorter; legs lacking apical tuft of setae or modification; genital complex as in Figure 2G.

**Description.***Male*. Body (Figure 2A) length 1.78 mm; color yellowish-brown.

Head (Figure 2B) sub-rectangular, wider than long, length from anterior margin of clypeus to head base 0.34 mm, width across eyes 0.41 mm; vertex sparsely and finely punctate, with distinct U-shaped sulcus connecting large, asetose foveae (dorsal tentorial pits), mediobasal carina short and thin, extending from head base anteriorly beyond level of posterior margin of eyes, lateral carina extending from base to posterior margin of antennal tubercle; posterolateral margin round; lacking frontal-clypeal ridge, clypeus with carinate and moderately raised anterior margin; ocular-mandibular carina complete, distinct. Venter with small gular foveae (posterior tentorial pits) in shared round opening, with shallow median carina extending from opening anteriorly to mouthpart. Compound eyes prominent, each composed of approximately 35 ommatidia. Antenna elongate, length 0.97 mm, lacking distinct club; antennomere 1 thick, subcylindrical, 2–7 elongate, 7 slightly wider than 6, 8 smallest, 9–11 each moderately enlarged, 11 largest, slightly shorter than 9 and 10 combined.

Pronotum (Figure 2B) approximately as long as wide, length 0.42 mm, width 0.41 mm, widest at middle; sides rounded, convergent basally, disc slightly convex, sparsely with minute punctures, median longitudinal sulcus from anterior l/3 to posterior l/8, with pair of lateral longitudinal sulci; broad antebasal sulcus connecting large, asetose lateral antebasal foveae; with small outer and inner pair of basolateral foveae. Prosternum with anterior part shorter than coxal part, with large lateral procoxal foveae; thin hypomeral ridge extending from base to middle of anterior part; margins of coxal cavities obviously carinate.

Elytra wider than long, length 0.61 mm, width 0.69 mm; each elytron with two moderately large, asetose basal foveae; thin discal stria extending posteriorly from outer basal fovea to apical 1/5 of elytral length; with small, angulate humeral prominence, subhumeral fovea present, carinate marginal stria extending from fovea to posterior margin of elytron.

Mesoventrite short, demarcated from metaventrite by transverse carina; median mesoventral foveae widely separated, in shared opening, lateral mesoventral foveae large, without internal fork, marginal stria complete. Metaventrite slightly prominent, weakly impressed at middle, with well-developed lateral mesocoxal and two lateral metaventral foveae; posterior margin with small and narrow split at middle.

Legs elongated; protibia with long apical tuft of setae; metafemur (Figure 2C) thick, strongly dilated at apical 2/5, dorsal surface of dilation with narrow cavity, with lamellar projection at bottom of cavity.

Abdomen widest at anterior margins of tergite 1 (IV), length of abdomen 0.46 mm, width 0.58 mm. Tergite 1 (IV) much longer than 2–4 (V–VII) combined, discal carina shot but distinct, thin basal sulcus separated by mediobasal and large basolateral foveae, lacking inner marginal carina, outer one thin, short, extending posteriorly from anterior margin of tergite 1 (IV) to basal 2/3 of tergite length; tergites 2–3 (V–VI) very short, tergite 4 (VII) twice as long as 2–3 (V–VI) combined in posterior view; tergite 5 (VIII) ovoid, convex, posterior margin roundly emarginate at middle. Sternite 2 (IV) with 2 pairs of mediobasal and one pair of basolateral foveae, with short lateral carina; midlength of sternites 3–5 (V–VII) short, lacking basolateral foveae; sternite 6 (VIII) transverse, posterior margin greatly emarginate; sternite 7 (IX) (Figure 2D) semi-membranous, apical portion narrowed and more strongly sclerotized, with small split at right.

Aedeagus (Figure 2E,F) 0.19 mm long, strongly asymmetric; median lobe with transversely extended basal capsule, broad foramen, and distinct basoventral projection; ventral stalk elongate, broadened at middle and apex; dorsal lobe in lateral view broad through entire length; parameres fused to single, broad membranous structure.

*Female*. Similar to male in external morphology; antenna slightly shorter; protibia lacking long apical tuft of setae, metafemur lacking large, swollen modification at apical 2/5; each compound eye composed of approximately 30 ommatidia. Measurements (as for male): body length 1.86 mm; length/width of head 0.37/0.42 mm, pronotum 0.43/0.42 mm, elytra 0.57/0.69 mm; abdomen 0.59/0.62 mm; length of antenna 0.99 mm; maximum width of genitalia (Figure 2G) 0.29 mm.

**Comparative notes.** The new species morphologically resembles *P. hasegawai* Nomura, 1991 in the similar form of the femoral modification, but can be readily separated by the different configuration of the aedeagus.

**Distribution.** China: Guangxi.

**Etymology.** The specific epithet refers to the strongly clavate metafemoral modification of the new species.

**Key to species of *Physomerinus* from China**

1 Median longitudinal sulcus relatively much longer, approaching both anterior and posterior margin of pronotum ……………………………. *P. schenklingi* (Raffray, 1912)
- Median longitudinal sulcus relatively much shorter, never approaching anterior margin of pronotum …………………………………………………………………………… 2
2 Metafemur with ventral oblique furrow …………………… *P. cruralis* (Raffray, 1914)
- Metafemur with cavity and projection/expansion ……………………………………. 3
3 Metafemur [Reference [6]: Figure 133A,D] hardly clavate, with deep excavation on dorsal surface and mushroom-shaped projection at bottom of excavation …………………………………………………………………. *P. pedator* (Sharp, 1883)
- Metafemur (Figure 2C) greatly clavate, with excavation on dorsal surface of expansion ……………………………………………………………………… *P. clavipes* sp. nov.

## 4. Discussion

The Nanling Mountain Range was identified as one of China’s biodiversity hotspots in terms of endemic plants and mammals [19]. Two studies on the local species richness of Argidae (Hymenoptera) and butterflies (Lepidoptera) indicated the insect fauna is predominated by Oriental elements [20,21]. Regardless, summarized knowledge regarding species diversity of most insect groups, including the subject of the present paper, the Pselaphinae, is largely unavailable. Prior to the present study, merely 16 species of Batrisini inhabiting several types of micro-habitats have been known to occur in the vast Nanling Mountain Area: two cavernicolous species each of *Araneibatrus* Yin and Li, 2010, and *Tribasodites* Jeannel, 1960, one myrmecophilous species each of *Dendrolasiophilus* Nomura, 2010 and *Songius* Yin & Li, 2010, and the others live freely in association with leaf litter layer or decomposing logs [22,23,24,25,26]. Aside from the two new species described here, we have recognized at least 100 additional species belonging to various groups of Batrisini from our sample, most of which was collected in the past three years and will be dealt with elsewhere. The true diversity of Pselaphinae from Nanling is difficult to evaluate at this moment due to the lack of taxonomic and distribution data, but the need in accelerating species description is evident when considering the ecosystems of the Nanling Mountain Range are fragile and sensitive to environmental change [27,28].

## Data Availability

Data sharing is not applicable to this article.
